# Levan Production by *Suhomyces kilbournensis* Using Sugarcane Molasses as a Carbon Source in Submerged Fermentation

**DOI:** 10.3390/molecules29051105

**Published:** 2024-02-29

**Authors:** Mariana González-Torres, Francisco Hernández-Rosas, Neith Pacheco, Josafhat Salinas-Ruiz, José A. Herrera-Corredor, Ricardo Hernández-Martínez

**Affiliations:** 1 Colegio de Postgraduados, Campus Córdoba, Carretera Federal Córdoba-Veracruz Federal Km 348, Congregación Manuel León, Municipio Amatlán de los Reyes, Veracruz 94946, Mexico; qfbmarianamc@gmail.com (M.G.-T.); fhrosas@colpos.mx (F.H.-R.); salinas@colpos.mx (J.S.-R.); jandreshc@colpos.mx (J.A.H.-C.); 2 Centro de Investigación y Asistencia en Tecnología y Diseño del Estado de Jalisco, Subsede Sureste, Mérida 97302, Mexico; npacheco@ciatej.mx; 3 CONAHCYT-Colegio de Postgraduados, Campus Córdoba, Carretera Federal Córdoba-Veracruz Federal Km 348, Congregación Manuel León, Municipio Amatlán de los Reyes, Veracruz 94946, Mexico

**Keywords:** levan, exopolysaccharides, carbon source, nitrogen source, *Suhomyces kilbournensis*

## Abstract

The valorization of byproducts from the sugarcane industry represents a potential alternative method with a low energy cost for the production of metabolites that are of commercial and industrial interest. The production of exopolysaccharides (EPSs) was carried out using the yeast *Suhomyces kilbournensis* isolated from agro-industrial sugarcane, and the products and byproducts of this agro-industrial sugarcane were used as carbon sources for their recovery. The effect of pH, temperature, and carbon and nitrogen sources and their concentration in EPS production by submerged fermentation (SmF) was studied in 170 mL glass containers of uniform geometry at 30 °C with an initial pH of 6.5. The resulting EPSs were characterized with Fourier-transform infrared spectroscopy (FT-IR). The results showed that the highest EPS production yields were 4.26 and 44.33 g/L after 6 h of fermentation using sucrose and molasses as carbon sources, respectively. Finally, an FT-IR analysis of the EPSs produced by *S. kilbournensis* corresponded to levan, corroborating its origin. It is important to mention that this is the first work that reports the production of levan using this yeast. This is relevant because, currently, most studies are focused on the use of recombinant and genetically modified microorganisms; in this scenario, *Suhomyces kilbournensis* is a native yeast isolated from the sugar production process, giving it a great advantage in the incorporation of carbon sources into their metabolic processes in order to produce levan sucrose, which uses fructose to polymerize levan.

## 1. Introduction

Exopolysaccharides are natural biopolymers that can be synthesized by some microorganisms such as fungi, bacteria, and yeast [1] and isolated from various sources such as extremophiles, halophiles, psychrophiles, and acidophiles, and their properties depend on the nature of the microorganism [2]. The principal advantage of microbial EPSs is their extracellular nature, and as a consequence, their recovery is relatively cheap compared with their intracellular counterparts [3]. The EPSs produced by microorganisms can be classified as hetero-polysaccharides and homo-polysaccharides. Hetero-polysaccharides are formed by the polymerization of different types of monosaccharides and their derivatives, whereas homo-polysaccharides consist of a single type of monosaccharide such as glucans, galactins, or fructans [4].

Fructans are fructose polymers, which include EPSs such as inulin and levan. Fructooligosaccharides (FOSs) are synthesized by fructosyltransferases (FTases; 2.4.1.9), which are a group of enzymes that have hydrolytic and transfructosyl activities. EPS production is carried out through the hydrolysis of sucrose and subsequent polymerization into FOSs. EPSs are well known for their properties and are used as sweeteners in the food and beverage industry and as prebiotics [5]. Also, they have been reported as safe for inclusion in food products because of their low caloric content as they are scarcely hydrolyzed by digestive enzymes and play an important role in reducing the levels of triglycerides and cholesterol. In addition, their production initially requires a high concentration of sucrose [6]. Nowadays, FOSs are gaining attention for their valuable attributes and economic potential in the sugar industry. Nonetheless, production processes with low-cost sources are needed in order to contribute to developing more sustainable and profitable processes [7]. The molecular weight of microbial fructan is usually hundreds of times higher than that of vegetable fructan as a result of the different enzymes responsible for fructan synthesis in microorganisms [8]. In the particular case of levan, levansucrase catalyzes the transfer of fructose units from sucrose to form β-2,6 glycosidic linkages, resulting in the formation of levan, which is primarily digested by the enzyme levanase, which breaks down the β-2,6 glycosidic linkages, releasing fructose as the main metabolite. It is generally synthesized by various microorganisms, e.g., bacteria such as *Zymomonas mobilis*, *Erwinia herbicola*, and *B. subtilis* or fungi such as *Aspergillus sydowii* and *Aspergillus versicolor*; reports of its production using yeast are scarce.

Levan is an EPS mainly composed of fructose units linked by β-(2,1) bonds [9] and has a wide range of applications. For example, it is used as an emulsifier, sweetener, and prebiotic in the food industry [10,11,12]; as a humectant and an antioxidant in the cosmetics industry [13,14]; and as an anti-inflammatory agent and an immunomodulator in the medical industry and pharmaceutical industries [15,16,17]. Levan is considered a novel EPS with a wide range of possible applications; for instance, it can be used as a thickener, stabilizer, fat substitute, or flavoring agent in dairy products because of its non-digestibility, non-toxicity, high stability, and solubility in water and oil; high water-holding capacity; and low intrinsic viscosity. Levan-based films have beneficial physicochemical and biological properties, such as biodegradability, edibility, and antibacterial and antifungal activity, and thus have good prospects as packing materials in the food, industrial, and medical sectors. On the other hand, levan possesses antioxidant, antitumor, antidiabetic, and immunomodulatory activities. In combination with levan’s promising characteristics in forming nanoparticles via self-assembly in water, levan-based nanoparticles have been proposed as prospective drug delivery carriers and cell proliferation agents [18]. Levan can be obtained from plant and microbial sources; however, microorganisms such as bacteria, fungi, and yeast have the ability to synthesize this EPS. Microbial levan is typically obtained through fermentation or enzymatic reactions using isolated enzymes, in which sucrose serves as both a principal carbon source and substrate, respectively. The optimal sucrose concentration for achieving maximum synthesis efficiency varies not only among different species but also often among different strains of microorganisms [19,20]. In the literature, there are various reports that indicate the capacity of some microorganisms to produce levan using sucrose as the only carbon source; among these, we can highlight *Bacillus subtilis* [21,22], *Lactobacillus reuteri* [23], and *Leuconostoc citreum* [24] and Gram-negative bacteria such as *Gluconobacter albidus* [25], *Brenneria goodwinii* [26], *Erwinia tasmaniensis* [27], and *Halomonas smyrnensis* [28]. Likewise, there are reports of the overexpression of the enzymes responsible for levan production; an example is the expression of the genes of *Rahnella aquatilis* [29] and *Leuconostoc mesenteroides* in *Saccharomyces cerevisiae* [30] and the genes of *B. subtilis* expressed in *Pichia pastoris* [31]. In the present work, levan was produced by *Suhomyces kilbournensis*, which has not been reported as an EPS producer. *Suhomyces* species have been discovered in association with insects, moths, flowers, moss, soil, and maize kernels [32]. Specifically, *Suhomyces kilbournensis* has been reported from one isolate obtained from uncharacterized soil in Mexico, and it has been isolated from maize kernels harvested in Illinois, USA. Moreover, *S. kilbournensis* has been reported as non-pathogenic. The growth of this yeast takes place via multilateral budding, and the cells occur singly and in pairs. Colony growth is white, opaque, creamy in texture, low with a slightly raised center, and bordered by pseudohyphae [33].

The production of EPSs depends on the synthesis of extracellular enzymes such as levan sucrose (EC 2.4.1.10), which is (regularly) responsible for the hydrolysis and transfructosylation reactions needed to synthesize levan using sucrose as a substrate [34,35]. Various reports show that the synthesis of both levan and the enzymes involved can be carried out by a diversity of microbes, among which, the most reported are bacteria, fungi, and archaea [36].

For the production of EPSs, it is important to characterize the production systems since the cultivation conditions, such as temperature, fermentation time, pH, and the sources and concentrations of carbon and nitrogen, are essential. FOS enzymes and EPS production require a fermentation system, which can be achieved through solid-state fermentation (SsF) or submerged-liquid fermentation (SmF). Both systems are well documented and can use agro-industrial byproducts to reduce manufacturing costs and obtain high yields of products. SsF can utilize agro-industrial byproducts, thus preventing negative environmental impact from waste accumulation. Nonetheless, SmF possesses several biotechnological advantages such as easy control of the fermentation parameters (pH, temperature, oxygen content), and it can be easily implemented at any scale [37]. Levan production has been carried out mainly using SmF since the biomass and EPS yields that have been obtained from it using various microorganisms are acceptable [38,39,40]; however, the development of processes using low-cost carbon sources is needed, as is the search for new microorganisms that enable increasing yields for the development of industrial processes that allow for low production costs [7,23]. Agro-industrial byproducts represent a promising alternative for the production of EPSs, FOS-producing enzymes, and FOS production. Byproducts, such as sugar cane molasses; beet molasses; agave syrups; fruit peels; some bagasse, such as sugar cane bagasse, coconut bagasse, corn bagasse, and agave bagasse; aguamiel; and coffee processing byproducts, are bioresources for levan-type FOS production [1,6]. Specifically, sugar cane molasses is the viscous liquid byproduct of the sugar extraction process from sugarcane juice and can have different chemical compositions depending on plant type, cultivation area conditions, plant maturity, and juice processing level. Molasses regularly contains sugar (content >43% in weight), polyphenols, vitamins, minerals, and ash. Owing to its nutrients, sugar cane molasses can be used as a carbon source for the production of EPSs and FOS enzymes [6]. In the literature, there are reports that demonstrate that agro-industrial byproducts such as beet molasses, sugar cane molasses, and syrup have been used as alternative carbon sources and have allowed for adequate microbial growth and EPS production via SmF [26,41]. Levan is an EPS with a high potential to be used in various industries given its physicochemical and functional characteristics [24,42]; however, to improve performance and quality, the design of a process allowing for large-scale, efficient, ecological, and profitable production is necessary [7,21]. Because of this, the objective of the present work was to evaluate the EPS production potential of the indigenous yeast strain *Suhomyces kilbournensis* under different process conditions.

## 2. Results

### 2.1. Kinetics of Exopolysaccharide Production by Suhomyces kilbournensis

**Figure 1:**
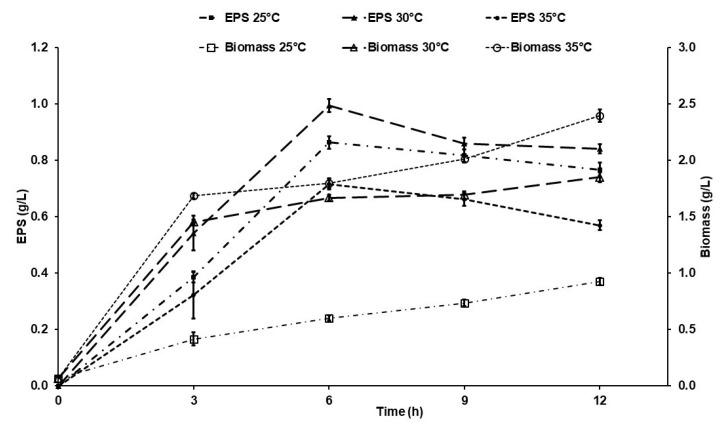
Effect of time on EPS production. The quantities of EPSs produced (g/L) were evaluated.

The results in Figure 1 show that the maximum EPS production using sucrose (40 g/L) as a carbon source was after 6 h of cultivation at all temperatures tested; however, the best performance occurred at 30 °C. The results show that the maximum production yields of EPSs at 25, 30, and 35 °C were 0.86, 0.99, and 0.71 g/L, respectively. In addition, after 9 h of cultivation, a decrease in productivity was observed at all temperatures tested. The time required for maximum EPS production (6 h) by *S. kilbournensis* was shorter than the time reported for other microorganisms such as *Bacillus subtilis* (20 h), *Tanticharoenia sakaeratensis* (35 h), *Leuconostoc citreum* BD1707 (96 h), *Gluconobacter albidus* (48 h), Halomonas smyrnensis (169 h), and *Acetobacter xylinum* NCIM2526 (122 h) [24,26,43,44,45,46]. On the other hand, Figure 1 shows that the highest biomass yield was present after 12 h of cultivation at 35 °C; however, the maximum production peak was not observed at any of the tested temperatures, indicating that EPSs and biomass production are not directly related. Sarilmiser et al. [45] indicated that the production of EPSs is associated with growth in some cases but not in others, depending on the microorganism used for this objective. The results of EPS production in the present investigation agree with what was reported by Abou-Taleb et al. [46], who reported that maximum EPS production occurred at 30 °C using *Bacillus lentus* V8 and at 25 to 30 °C using *Leuconostoc citreum* BD1707 [26]. It is important to mention that temperature is an important parameter that affects microbial growth, intracellular metabolic processes, and EPS yield [26,47,48]. There are reports that indicate that the optimal temperature for the enzymatic activity of a levansucrase produced by *B. subtilis* is between 30 and 37 °C [36]. These extracellular enzymes are responsible for the synthesis of EPSs such as levan. Since production occurs regularly in a microbial system, it is important to control the culture conditions, as they influence both the metabolism of the microorganism and the catalytic activity of the enzyme [27]. Furthermore, the optimal temperature of enzymatic activity is the fundamental condition since it can ensure the efficient synthesis of EPSs [32].

The results in Figure 1 show a direct relationship between temperature and growth; as the temperature increases, biomass production increases until it has a yield of 1.79 g/L at 35 °C. Similar results were found by Jadhav et al. [32], who reported that *S. kilbournensis* presents its optimal growth between 30 and 37 °C. Since, in the present work, the maximum EPS production was obtained at 30 °C (0.99 g/L), subsequent experiments were carried out at 30 °C.

### 2.2. Effect of pH on Growth and Production of Exopolysaccharides

**Figure 2:**
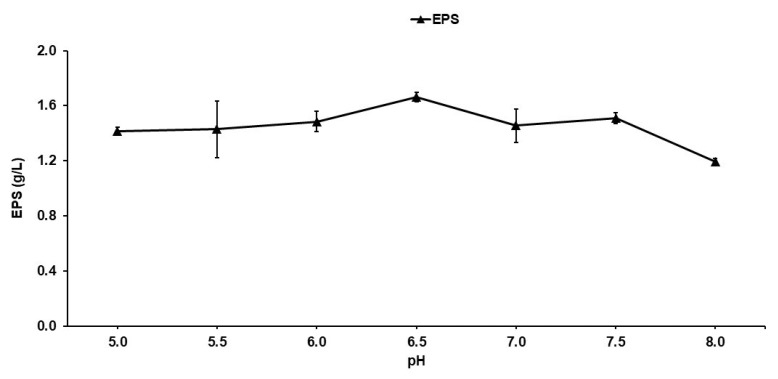
Effect of pH on the production of EPSs by *S. kilbournensis*.

The results of EPS production at different pH values (Figure 2) demonstrate that EPS concentration increased as pH increased, presenting a maximum production of 1.66 g/L at pH 6.5; however, after this pH, the production of EPSs decreased. In accordance with these results, the following experiments (the effects of nitrogen and carbon sources on EPS production) were conducted with an initial pH of 6.5. The EPS production profile during SmF at different initial pH values may be because EPS synthesis depends on the action of an extracellular enzyme that has a catalytic response to pH changes, directly impacting EPS yields [19]. As shown in Figure 2, the highest EPS concentration was obtained in the production system implemented in the present investigation when the initial pH of the SmF was adjusted to 6.5. The results of the statistical analysis indicated that EPS production did not show significant differences at the different initial values of pH tested. This result was similar to the results reported by Belgith et al. [49], who indicated that pH is very important for the synthesis of EPSs and obtained the best result at a pH value of 6.5, probably because of the synthesis of levansucrase being improved at these pH values when *Bacillus* spp. were used as an inoculum. Furthermore, this study reported that this enzyme was responsible for fructose polymerization in the synthesis of the EPSs. Likewise, Mummaleti et al. [22] reported similar results (pH 6.8) using *Bacillus subtilis* as an inoculum. This agreed with the results reported by Öner et al. [50], who reported the highest levan production at pH 6.0 using *B. methylotrophicus* and that the optimal pH for levansucrase activity was between 5.0 and 6.5. Likewise, in a fructosylated EPS (levan) production system using *Gluconobacter albidus*, it was reported that the levan produced at pH 6.5 maintained a constant size and molecular weight [44]. It is important to mention that reports indicated that transfructosylation activity can occur in slightly acidic conditions at a pH range of 4.0–6.5 [51].

### 2.3. Effect of Nitrogen Source on Exopolysaccharide Production

**Figure 3:**
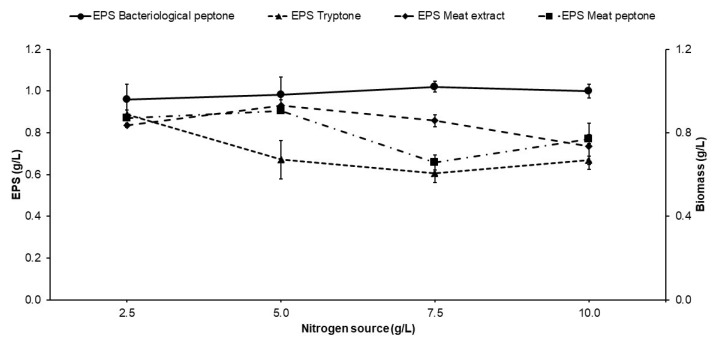
Effect of nitrogen source on EPS production by *S. kilbournensis*.

The results for the effect of the nitrogen source on EPS production can be observed in Figure 3. The effect of the nitrogen source on the metabolism of *S. kilbournensis* was determined by evaluating four nitrogen sources: bacteriological peptone, meat peptone, tryptone, and meat extract. The results of the statistical analysis of EPS production showed a significant difference when bacteriological peptone was used at a concentration of 7.5 g/L, followed by concentrations of 0.93 and 0.90 g/L when meat extract and meat peptone were used at a concentration of 5 g/L, respectively, and a yield of 0.88 g/L when tryptone was used at a concentration of 2.5 g/L. Since the maximum EPS production was obtained at 7.5 g/L of bacteriological peptone, subsequent experiments were carried out under those conditions. The results obtained in the present work are consistent with those reported by Srikanth et al. [43], who reported a maximum yeast yield of 1.14 g/L produced with *Acetobacter xylinum* NCIM2526 using 10 g/L of bacteriological peptone. The findings are also similar to the results obtained by Mamay et al. [52], who obtained the best results when they used bacteriological peptone as a carbon source with *Bacillus licheniformis* BK AG1. In the literature, some reports indicate that the nitrogen source used for EPS production can have negative or positive effects on production depending on the microorganism used. In the case of peptone and yeast extract, there are several reports that indicate positive effects, attributable to the content of polypeptides, vitamins, and minerals that favor the metabolism of the microorganism for EPS production [53]. In the particular case of *S. kilbournensis*, there are reports that indicate that the sources of organic nitrogen can easily influence its metabolism, which agrees with the results obtained in the present study. Likewise, it has been reported that *S. kilbournensis* cannot assimilate nitrate [32,54].

### 2.4. Effect of Carbon Source on Exopolysaccharide Production

**Figure 4:**
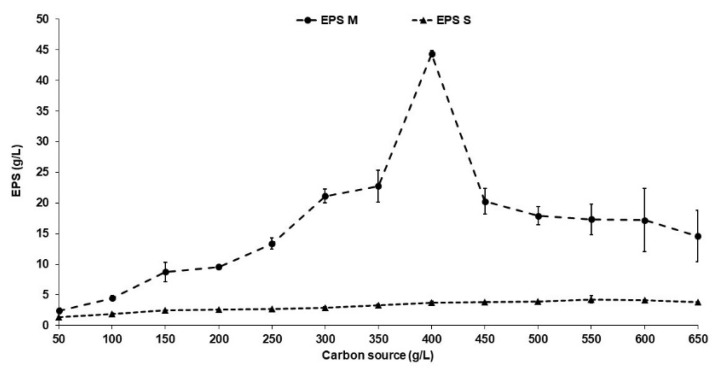
Effect of carbon source on EPS production by *S. kilbournensis*. S: EPS produced with sucrose; M: EPS produced with molasses.

In order to determine the effect of the carbon source on the production of EPSs, SmF was realized using sucrose and molasses as a carbon source and bacteriological peptone as a nitrogen source. The results shown in Figure 4 indicate that the highest EPS concentration (44.33 g/L) was obtained when 400 g/L of molasses was used as a carbon source, while the maximum yield was 4.46 g/L (a yield of 10 times more) when sucrose was used at a concentration of 550 g/L. Likewise, Figure 4 shows a direct relationship between the molasses concentration and EPS production; when the molasses concentration was increased, the EPS concentration increased until reaching 400 g/L, and after this, it decreased proportionally. On the other hand, when sucrose was used as a carbon source, the behavior was similar to when molasses was used; however, the yields were 10 times lower than those obtained with molasses. According to the statistical analyses, molasses at 400 g/L showed the highest production, and this carbon source has the advantage of being the cheapest feedstock, reducing the production costs of EPSs.

The results obtained in this research are similar to other reports that indicate that high concentrations of a carbon source can improve EPS production, particularly for levan [26,53]. Likewise, the results are consistent with the results obtained by Zhang et al. [55], who reported a maximum EPS yield when 300 g/L of sucrose was used as a carbon source with *Bacillus methylotrophicus*. Furthermore, the EPS yield decreased significantly above this sucrose concentration, probably because of the increase in viscosity and an enzymatic inhibition that consequently impacted EPS synthesis [19,41]. On the other hand, the use of recombinant yeasts for levan production has been reported; e.g., Ko et al. [29] reported a levansucrase from *Rahnella aquatilis* expressed in *Saccharomyces cerevisiae*, obtaining yields of 3.17 g/L/h. Likewise, by expressing a fusion enzyme between endolevanase from *B. licheniformis* and levansucrase from *B. subtilis* in *Pichia pastoris*, yields of 0.82 g/L/h were obtained [5]. Also, Shang et al. [56] reported that the levansucrase enzyme from *Zymomonas mobilis* was expressed in *Saccharomyces cerevisiae* EBY100, obtaining a levan production yield of 1.42 g/L/h. Furthermore, there was a significant increase in EPS production using molasses as a carbon source, probably because molasses contains high levels of sucrose, nitrogen compounds, and trace elements that promote microbial growth and enhance EPS synthesis [41,57].

### 2.5. Characterization of Exopolysaccharides with Fourier-Transform Infrared Spectroscopy (FT-IR)

**Figure 5:**
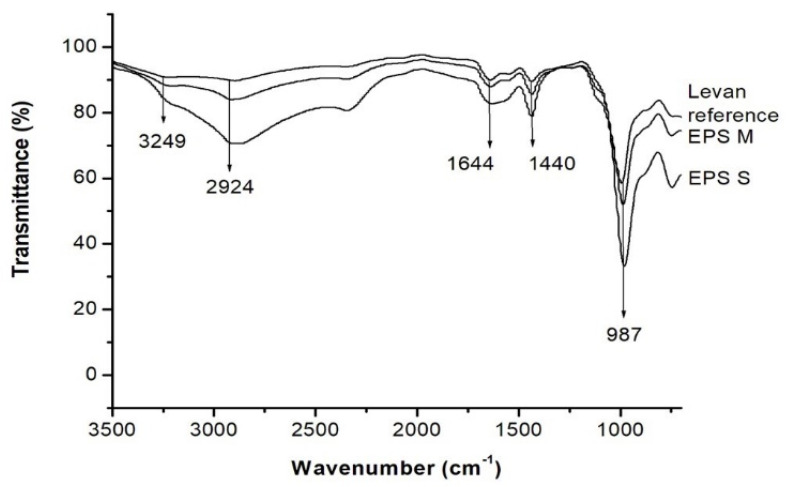
FT−IR of the recovered EPSs, EPS M, EPS S, and the levan reference.

Fourier-transform infrared spectroscopy was used to determine the structure of EPSs produced by *S. kilbournensis* (Figure 5). A strong band of OH stretching was observed at 3249 cm^−1^. The bands within the region of 3600–3200 cm^−1^ were due to OH vibration [58], and the band at 2924 cm^−1^ specifies CH bending. The region in the range of 3000–2800 cm^−1^ indicates the stretching vibration of CH and confirms the presence of fructose [22]. The spectrum band at 1644 cm^−1^ indicates carbonyl stretching [43], and the peak at 1440 cm^−1^ corresponds to the CH vibration [22]. The band at 987 cm^−1^ corresponds to the vibration of the glycosidic bond, and the region in the range of 1200–900 cm^−1^ is characteristic of polysaccharides because the ring vibrations overlap with the vibration of the COC glycosidic bond and the stretching vibration of the COH side groups [22,43]. The EPSs produced by *S. kilbournensis* showed bands corresponding to levan.

## 3. Materials and Methods

### 3.1. Microorganisms and Growth Conditions

EPS production was carried out using *S. kilbournensis.* This strain was isolated from a regional sugar mill, specifically from a sugar mill manufacturing honeydew [59]. The strain was grown in a culture medium composed of (g/L) yeast extract (BD Bioxon^®^, Mexico) (10), peptone (Hycel^®^, Mexico) (20), dextrose (BD Bioxon^®^, Mexico) (10), and agar (BD Bioxon^®^, Mexico) (15) [60] and was kept at 30 °C for 24 h. The strain was preserved in a 30% (*v*/*v*) glycerol solution at 4 °C until use.

### 3.2. Inoculum Preparation

For inoculum preparation, *S. kilbournensis* was inoculated in SmF in Luria broth medium supplemented with (g/L) sucrose (BD Bioxon^®^, Mexico City, Mexico) (6), peptone (Hycel^®^, Mexico City, Mexico) (1), (NH_4_)_2_SO_4_ (Meyer^®^, Mexico City, Mexico) (0.2), KH_2_PO_4_ (Fermont™, Mexico City, Mexico) (0.1), and MgSO_4_·7H_2_O (J.T. Baker^®^, Mexico) (0.1) with an adjusted initial pH of 6.8 and was maintained at 30 °C at 150 rpm for 24 h. For biomass recuperation, the culture was centrifuged at 3500× *g* for 15 min; then, the pellet was resuspended in distilled water, and the number of cells was determined using a Neubauer chamber. The suspension obtained was considered the SmF inoculum for the production of EPSs and was stored at 4 °C until use.

### 3.3. Production of Exopolysaccharides

The production of EPSs was carried out via SmF in glass containers of uniform geometry with a capacity of 170 mL by adding 75 mL of a culture medium [43]. The medium was composed of (g/L) sucrose (40), bacteriological peptone (10), (NH_4_)_2_SO_4_ (1), KH_2_PO_4_ (1), and MgSO_4_·7H_2_O (1) with an initial adjusted pH of 6 and an inoculum concentration adjusted at 1 × 10^6^ CFU/mL and maintained at 30° C with constant stirring at 150 rpm. Sampling was carried out at regular intervals of 3, 6, 9, and 12 h for the quantification of biomass and EPSs [41].

### 3.4. Recovery and Purification of Exopolysaccharides

The EPSs produced with SmF were recovered for fermented culture boiled for 30 min, followed by centrifugation at 3500× *g* for 15 min. The supernatant obtained was subjected to a second boiling treatment for 5 min, followed by a pH adjustment to 10 using 1 M of KOH. Finally, the EPSs were precipitated by adding chilled ethanol (80% *v*/*v*) at a ratio of 2:1 (*v*/*v*). The mixture was maintained by stirring at 4 °C overnight, followed by the addition of CaCl_2_ (1%) with constant stirring for 20 min. The precipitate obtained was recovered via centrifugation at 3500× *g* for 20 min, and the pellet obtained was washed with a 1.5 volume of chilled ethanol (80% *v*/*v*) [43] and a lyophilizer (Labconco™ FreeZone™ 4.5, Kansas City, MO, USA) for subsequent analyses.

### 3.5. Effect of Different Variables on Exopolysaccharide Production via SmF

The effects of temperature, pH, and carbon and nitrogen sources on EPS production in SmF were determined, evaluating the effect of individual parameters.

#### 3.5.1. Effect of pH on the Production of Exopolysaccharides

To determine the effect of pH on EPS production, the initial pH of the culture medium was adjusted to different initial pH values between 5.0 and 8.0 [55].

#### 3.5.2. Effect of Temperature on the Production of Exopolysaccharides

The effect of temperature on EPS production was determined, maintaining the SmF at 25, 30, and 35 °C [58].

#### 3.5.3. Effect of Different Nitrogen Sources on the Production of Exopolysaccharides

The effect of four nitrogen sources (bacteriological peptone, meat peptone, meat extract, and tryptone) on EPS production in SmF was evaluated [43].

#### 3.5.4. Effects of Carbon Source and Concentration in Exopolysaccharide Production

The effects of the source and concentration of carbon on the production of EPSs were determined. The SmF was carried out with sucrose and molasses at different concentrations (50, 100, 150, 200, 250, 300, 350, 400, 450, 500, 550, 600, and 650 g/L) [45,55].

### 3.6. Structural Characterization of the Exopolysaccharides

The EPSs produced using SmF were characterized with FT-IR using Thermo Scientific Nicolet 8700 equipment (with a resolution of 16 cm^−1^) in attenuated total reflection (ATR) sampling mode from 650 to 4000 cm^−1^. For the analysis, a levan standard from *Erwinia herbicola*, inulin from Dahlia tubers, and dextran from *Leuconostoc mesenteroides* (Sigma-Aldrich^®^, St. Louis, MA, USA) were used [59].

### 3.7. Statistical Analysis

The data were subjected to analysis of variance (ANOVA) using RStudio version 2023.09.1. The means were compared using the Tukey test, and significance was defined at *p* < 0.05.

## 4. Conclusions

In this study, levan of the novel strain *Suhomyces kilbournensis* was produced in a short time (6 h) with the best parameter results in SmF (30 °C; pH 6.5; and bacterial peptone, 7.5 g/L, and molasses, 400 g/L, as nitrogen and carbon sources, respectively). The cultivation conditions showed that the pH, temperature, and nitrogen source are important parameters for levan production; however, the carbon source and its concentration were the most relevant parameters for improving levan production.

## Data Availability

Data are contained within the article.
